# Supplementary material for: Complexome profiling of the Chlamydomonas psb28 mutant reveals TEF5 as an early PSII assembly factor
Source: Plant Cell. 2025 Mar 17;37(6):koaf055. doi: 10.1093/plcell/koaf055 (PMC12164590; doi:10.1093/plcell/koaf055)
Supplement: koaf055_Supplementary_Data [file koaf055_supplementary_data.zip › psb28_interactive.zip!/plots/intensity/qualityPlots/peptides/PLAP2_Cre03.g188650.t1.2.html]

### Cre03.g188650.t1.2

Trivial name: PLAP2  
SeqCoverage [median]: 11.10  
Mass: 24.63 kDa  
  
 

| Sequence | MissedCleavage | Mass [Da] | wtIntensity | muIntensity | Score |
| --- | --- | --- | --- | --- | --- |
| GASATADDKADVER | 1 | 1404.65 | 477.71 | 608.79 | 80.47 |
| LINITAPPAAK | 0 | 1107.67 | 0.00 | 338.75 | 64.71 |
|  |  |  | 477.7 | 947.5 |  |
